# Discovery of new boron-rich chalcogenides: orthorhombic B_6_X (X=S, Se)

**DOI:** 10.1038/s41598-020-66316-y

**Published:** 2020-06-09

**Authors:** Kirill A. Cherednichenko, Vladimir A. Mukhanov, Zhenhai Wang, Artem R. Oganov, Aleksandr Kalinko, Iurii Dovgaliuk, Vladimir L. Solozhenko

**Affiliations:** 1 LSPM–CNRS, Université Paris Nord, Villetaneuse, 93430 France; 2 Skolkovo Institute of Science and Technology, Skolkovo Moscow Region, 143026 Russia; 3 Nanjing University of Posts and Telecommunications, Nanjing, Jiangsu 210003 China; 4 Moscow Institute of Physics and Technology, Dolgoprudny City, Moscow Region, 141700 Russia; 5 School of Materials Science, Northwestern Polytechnical University, Xi’an, 710072 China; 6 Institute of Solid State Physics, University of Latvia, Riga, LV-1063 Latvia; 7 Universität Paderborn, Naturwissenschaftliche Fakultät, Paderborn, 33098 Germany; 8 European Synchrotron Radiation Facility, Grenoble, 38043 France

**Keywords:** Structure prediction, Structure of solids and liquids, Mechanical properties

## Abstract

New boron-rich sulfide B_6_S and selenide B_6_Se have been discovered by combination of high pressure – high temperature synthesis and *ab initio* evolutionary crystal structure prediction, and studied by synchrotron X-ray diffraction and Raman spectroscopy at ambient conditions. As it follows from Rietveld refinement of powder X-ray diffraction data, both chalcogenides have orthorhombic symmetry and belong to *Pmna* space group. All experimentally observed Raman bands have been attributed to the theoretically calculated phonon modes, and the mode assignment has been performed. Prediction of mechanical properties (hardness and elastic moduli) of new boron-rich chalcogenides has been made using *ab initio* calculations, and both compounds were found to be members of a family of hard phases.

## Introduction

Development of modern industry requires more new materials with exceptional physical and chemical properties. Searching for such materials becomes a central challenge of modern materials science. The discoveries of fullerene, carbon nanotubes and graphene unveiled that unusual crystal structures give access to the unique properties.

Boron-rich compounds are materials possessing such unusual structures. The B_12_ *closo*-clusters are the common feature of these compounds. Almost all boron-rich solids may be considered as a combination of ‘electron deficient’ B_12_-icosahedral units (36 valence electrons over 48 bonding orbitals) and various interstitial atoms (from nonmetals to metals)^1,2^. The multicenter metal-like bonding system within the B_12_ icosahedra and strong covalent bonds between B_12_ *closo*-clusters and interstitial atoms makes boron-rich compounds extremely stable, which leads to high melting temperatures, chemical inertness and outstanding mechanical properties^1–4^. A change of the interstitial atoms makes it possible to considerably vary the properties (e.g. bulk moduli variation of α-rhombohedral boron (α-B_12_) and isostructural boron-rich compounds: B_4_C, B_12_O_2_, B_13_N_2_, B_12_P_2_^5,6^). Thus, a detailed study of already existing materials and exploration of new boron-rich compounds are of great importance and draw considerable attention in experiment and theory.

In the present work two new boron-rich chalcogenides were synthesized under high pressure–temperature conditions. Their crystal structures were found by *ab initio* crystal structure prediction, which allowed us to perform Rietveld refinement of the experimental X-ray diffraction (XRD) patterns. The Raman spectra of both boron-rich chalcogenides were acquired at ambient conditions, and the observed Raman bands were assigned to the specific phonon modes.

## Results and Discussion

According to the energy-dispersive X-ray spectroscopy data, the elemental composition of synthesized chalcogenides is 86.1(7) at% B and 13.9(7) at% S for boron sulfide, and 86(1) at% B and 14(1) at% Se for boron selenide, so the stoichiometry of both compounds is B_6_X (X = S, Se).

### Crystal structure of new boron-rich sulfide and selenide

**Figure 1:**
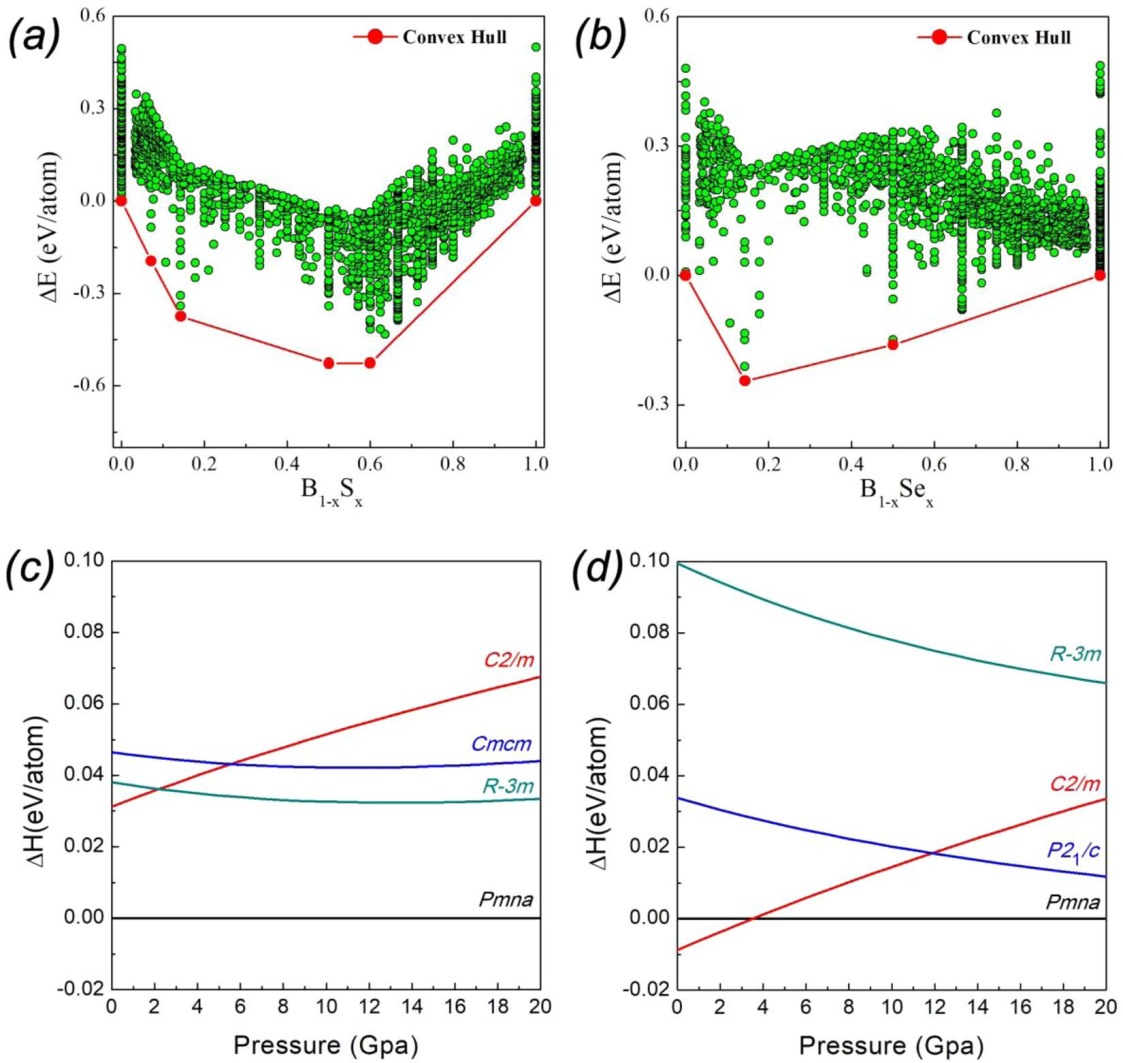
Convex hull of B-S (**a**) and B-Se (**b**) from variable-composition USPEX calculations at 20 GPa. Enthalpy difference (∆H) between stable/metastable B_6_S (**c**) and B_6_Se (**d**) structures in the 0–20 GPa pressure range.

The crystal structures of new phases were predicted using the USPEX algorithm. We found that at 20 GPa the following boron-rich chalcogenides are thermodynamically stable (see thermodynamic convex hulls in Fig. 1a,b): B_6_S, BS, B_2_S_3_ for boron sulfides, and B_6_Se and BSe for boron selenides. The computed enthalpies of the lowest-enthalpy structures as a function of pressure are shown in Fig. 1c,d: B_6_S is stable in the *Pmna* structure at least in the 0–20 GPa pressure range, whereas the structure of B_6_Se with *Pmna* space group is stable in the 4–20 GPa range.

**Figure 2:**
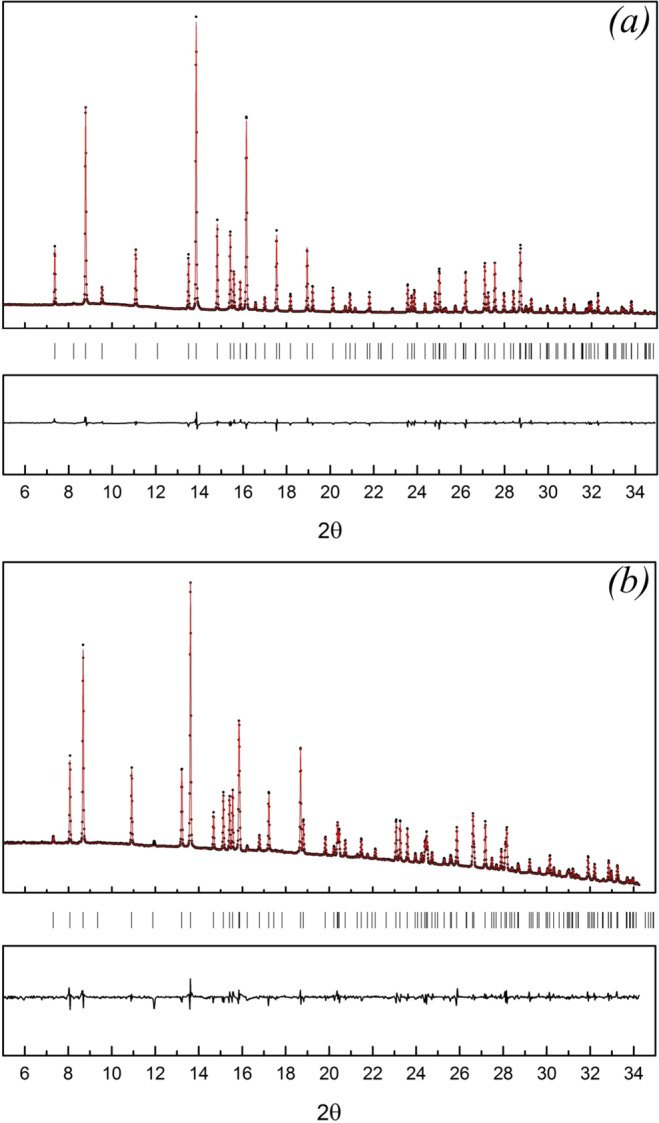
Rietveld full profile refinement of powder X-ray diffraction patterns of *o*-B_6_S (**a**) and *o*-B_6_Se (**b**).

Theoretically predicted crystal structures of boron-rich chalcogenides were further used as starting models for Rietveld refinement of the powder X-ray diffraction patterns taken at ambient conditions (Fig. 2). The backgrounds of both diffraction patterns were approximated by a 5-order polynomial. The final reliability factors *R*_*wp*_ converged to 5.0% (see Fig. 2a) and 5.8% (see Fig. 2b) indicate the excellent refinement level and, thus, confirming the correctness of the structures found with USPEX algorithm. The refined lattice parameters of boron-rich sulfide and selenide are presented in Table 1.

**Table 1:** Unit cell parameters (*a*_0_, *b*_0_, *c*_0_) and predicted mechanical properties of *o*-B_6_X (X = S, Se): bulk modulus (*B*_0_), shear modulus (*G*), Young’s modulus (*E*), Poisson’s ratio (*v*), Vicker’s hardness (*H*_V_) and fracture toughness (*K*_Ic_).

	*o*-B_6_S (*Pmna*)			*o*-B_6_Se (*Pmna*)		
	Exp.	VASP	CRYSTAL17	Exp.	VASP	CRYSTAL17
*a*_0_, Å	5.8170(1)	5.8307	5.8139	5.9463(1)	5.9684	5.9359
*b*_0_, Å	5.3025(1)	5.3202	5.2918	5.3579(1)	5.3802	5.3416
*c*_0_, Å	8.2135(1)	8.2072	8.2026	8.3824(1)	8.3809	8.3631
V_0_, Å^3^	253.34(1)	254.59	252.36	267.06(1)	269.12	265.17
*B*_0_, GPa	—	146	151	—	137	144
*G*, GPa	—	138	—	—	135	—
*E*, GPa	—	315	—	—	304	—
*v*	—	0.14	—	—	0.13	—
*H*_V_, GPa	—	31^*a*^ 24^*b*^	—	—	32^*a*^ 24^*b*^	—
*K*_Ic_, MPa·m^1/2^	—	2.1^*c*^ 1.5^*d*^	—	—	2.0^*c*^ 1.2^*d*^	—

^*a*^Chen model^45^.

^*b*^Mazhnik-Oganov model^46^.

^*c*^Niu-Oganov model^47^.

^*d*^Mazhnik-Oganov model^46^.

**Figure 3:**
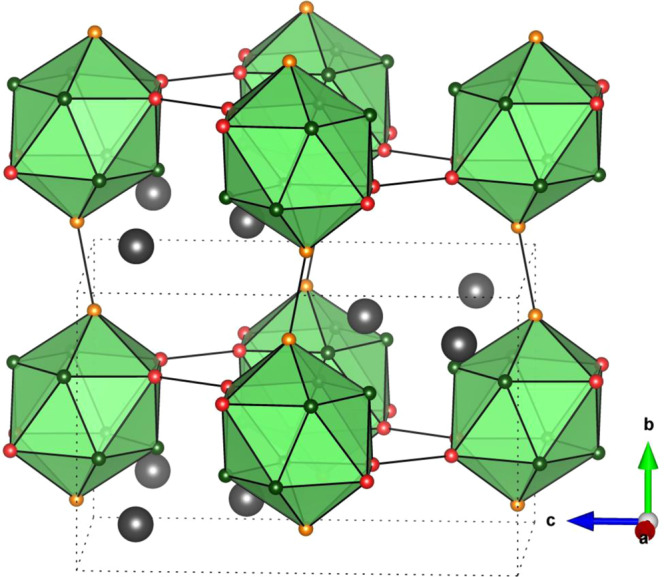
Crystal structure of new orthorhombic phases of boron-rich sulfide and selenide, *o*-B_6_X (B_12_-units are presented by green icosahedral polyhedral; polar B1; equatorial B2; and equatorial B3 & B4 atoms are marked by orange, red and green balls, respectively; X = S, Se atoms are shown as large grey balls).

The unit cell of both boron-rich chalcogenides contains 24 boron atoms in four independent (*4 h* and *8i*) Wyckoff positions and 4 sulfur/selenium atoms placed in one independent (*4 h*) Wyckoff position. Since all boron atoms constitute B_12_ clusters their total atom site occupancies were fixed to 1.0 by default. The total S1 and Se1 sites occupancies were found to be 0.925 and 0.952, respectively. The details of atomic structure of both compounds are presented in Table S1. Considering the occupancies of S1 and Se1 sites are close to 1, the stoichiometry of new orthorhombic boron-rich sulfide and selenide may be presented as “B_6_X”, where X is S or Se. It should be underlined that the attempt to replace S and Se atoms by B atoms resulted in a large mismatch and high *R*_*wp*_ values. For convenience and in order to avoid any confusion with previously reported hexagonal boron-rich chalcogenides (e.g. B_12_S_2-*x*_^7,8^ and B_12_Se_(2-*x*)_B_*x*_^9^) further we will call the new boron-rich sulfide and selenide as “*o*-B_6_S” and “*o*-B_6_Se” (where “*o*” indicates the orthorhombic symmetry). The unit cell of *o*-B_6_X (where X = S, Se) is presented in Fig. 3. The X-ray densities of *o*-B_6_S and *o*-B_6_Se were found to be 2.54 g/cm^3^ and 3.58 g/cm^3^, respectively which is in good agreement with values predicted *ab initio* using USPEX (2.53 g/cm^3^ and 3.55 g/cm^3^) and CRYSTAL17 (2.58 g/cm^3^ and 3.66 g/cm^3^).

Among all experimentally obtained nonmetal boron-rich compounds only *o*-B_6_S and *o*-B_6_Se have the orthorhombic structure (with exception of B_6_Si (*Pnnm*)^10^ and B_3_Si (*Imma*)^11^). The distribution/packing of the B_12_ *closo*-clusters in *o*-B_6_X (X = S, Se) unit cells may be described as base-centered (Fig. 3) similar to that in B_3_Si. One slightly distorted B_12_-icosahedron in *o*-B_6_S and *o*-B_6_Se is linked with six others. The lengths of intra-icosahedral B–B bonds vary from 1.7294 Å to 1.8987 Å in *o*-B_6_S and from 1.7077 Å to 1.9009 Å in *o*-B_6_Se, whereas the inter-icosahedral bond lengths in *o*-B_6_S and *o*-B_6_Se are: 1.6949 Å (B1–B1), 1.7448 Å (B2–B2), and 1.7511 Å (B1–B1), 1.8004 Å (B2-B2), respectively. One sulfur/selenium atom is linked with three closest icosahedra: S1–B4 (1.8884 Å), S1–B3 (1.8586 Å), Se1–B3 (1.9623 Å) and Se1–B4 (2.0128 Å).

The predicted lattice parameters and mechanical properties, as well as atomic positions of new boron-rich chalcogenides are presented in Table 1 and Table S1. Both phases are considerably more compressible (*B*_0_ values estimated by VASP and CRYSTAL17 are in good agreement) and less hard than γ-B_28_^12,13^, α-rhombohedral boron^14–16^ and isostructural boron-rich compounds^5,6,17–21^.

### Raman spectra of new boron-rich sulfide and selenide

*o*-B_6_S and *o*-B_6_Se have 28 atoms in the unit cell, thus, 84 normal modes are expected. According to the symmetry analysis, the acoustic and optical modes of *o*-B_6_X (where X = S or Se) at Г point can be presented as follows:

$${\Gamma }_{{\rm{acoustic}}}={{\rm{B}}}_{{\rm{1u}}}+{{\rm{B}}}_{2{\rm{u}}}+{{\rm{B}}}_{3{\rm{u}}}$$

$${\Gamma }_{{\rm{optic}}}=12{{\rm{A}}}_{{\rm{g}}}+9{{\rm{A}}}_{{\rm{u}}}+9{{\rm{B}}}_{1{\rm{g}}}+11{{\rm{B}}}_{1{\rm{u}}}+9{{\rm{B}}}_{2{\rm{g}}}+11{{\rm{B}}}_{2{\rm{u}}}+12{{\rm{B}}}_{3{\rm{g}}}+8{{\rm{B}}}_{3{\rm{u}}}$$

11B_1u_ + 11B_2u_ + 8B_3u_ are IR-active modes; 12A_g_ + 9B_1g_ + 9B_2g_ + 12B_3g_ are Raman-active modes; others are silent modes.

**Figure 4:**
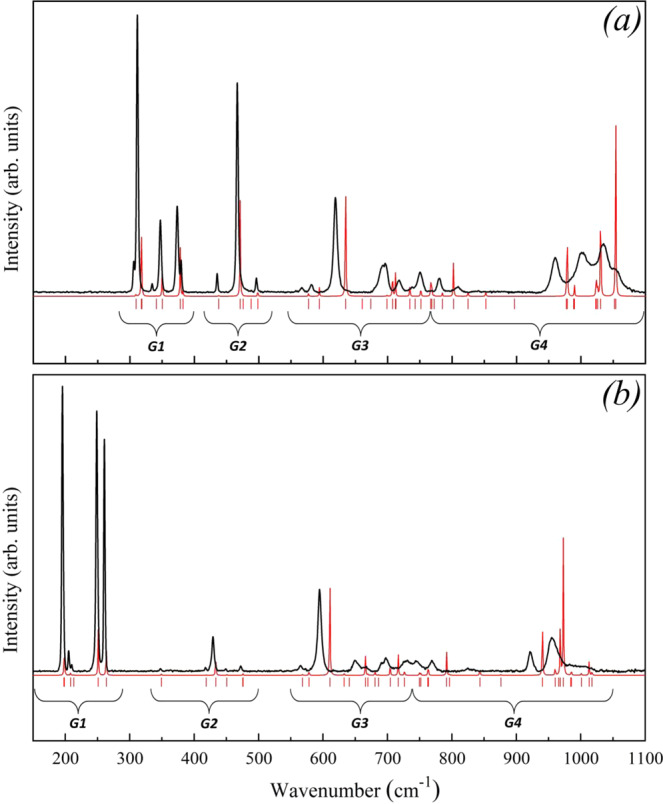
The experimental (black) and calculated by CRYSTAL17 (red) Raman spectra of *o*-B_6_S (**a**) and *o*-B_6_Se (**b**). The red dashes show all predicted Raman active phonon modes.

Raman spectra of *o*-B_6_S and *o*-B_6_Se were measured in the 100–2500 cm^−1^ frequency range, however, all bands were observed in the 150–1100 cm^−1^ region (Fig. 4). The Raman spectra of *o*-B_6_S and *o*-B_6_Se resemble the Raman spectra of α-B_12_^22,23^ and γ-B_28_^24^ and other boron-rich compounds^25–28^. The most intense and narrow bands are situated in the low-frequency region (<500 cm^−1^ for *o*-B_6_S and <400 cm^−1^ for *o*-B_6_Se), whereas the less intense and broad bands and band groups are concentrated in the high-frequency region.

The CRYSTAL17 and VASP calculated Raman spectra of both compounds (at T = 0 K) are presented in Fig. 4 and Fig. S1, respectively. The theoretically predicted Raman active phonon modes (ω_*t*_^C^ and ω_*t*_^V^, for CRYSTAL17 and VASP, respectively), experimentally observed Raman bands and overlapped band groups (ω_0_) of *o*-B_6_S and *o*-B_6_Se are listed in Table S2. The theoretical and experimental data were found to be in a good agreement. The average error on individual modes being less than 1.5% for *o*-B_6_S (with a maximum error of 2.8%; mode at 780 cm^−1^) and 1.2% for *o*-B_6_S (with a maximum error of 2.7%; mode at 594 cm^−1^). Good agreement between theory and experiment (also observed in our previous Raman studies^29,30^) gave us confidence in the predictive power of our *ab initio* calculations for mode assignment (see Table S2).

The predicted phonon modes were confidently associated with the corresponding atomic movements in *o*-B_6_S and *o*-B_6_Se unit cells with help of visualization procedure built in MOLDRAW software^31^. Taking into account that normal modes of boron-rich chalcogenides with such complicated structure may incorporate various simultaneous atomic movements, we distinguished the most distinct ones only for convenience of the description. As one can see in Fig. 4, the Raman bands of both spectra were divided onto four groups (*G1*–*G4*).

The “*G1*” contains the low frequency modes (280–400 cm^−1^ for *o*-B_6_S and 150–280 cm^−1^ for *o*-B_6_Se) corresponding to symmetric and antisymmetric oscillations (e.g. rocking, twisting, wagging) of S/Se atoms and the corresponding B_12_–icosahedral units distortions.

The Raman bands of group “*G2*” (420–520 cm^−1^ for *o*-B_6_S and 330–500 cm^−1^ for *o*-B_6_Se) are associated with various tilting oscillations of the whole B_12_ units around different crystallographic [100], [010] and [001] directions (rocking and wagging of the equatorial and polar boron atoms of one B_12_-unit). Unlike Se atoms, the oscillations of S atoms were found rather significant in some “*G2*” modes. This phenomenon can be easily explained by the atomic mass difference of S and Se atoms.

The middle-frequency modes in “*G3*” (550–760 cm^−1^ for *o*-B_6_S and 550–740 cm^−1^ for *o*-B_6_Se) correspond, first of all, to different vibrations of the equatorial B atoms (B2 - B4) leading to stretching of the intra-icosahedral B–B bonds, rotations of the B1–B1 and B2–B2 inter-icosahedral bonds and rotations, twisting and “umbrella” oscillations of the S–(B)_3_ structural elements (three B atoms belong to three different B_12_-units).

The “*G4*” group contains the high-frequency modes (760–1100 cm^−1^ for *o*-B_6_S and 740–1050 cm^−1^ for *o*-B_6_Se) described by oscillations of the equatorial and polar boron atoms of B_12_ units leading to stretching of the inter-icosahedral bonds (B–X, B2–B2, B1–B1). For instance, in both spectra the two phonon modes with the highest frequencies correspond to the oscillations of the polar B1 atoms and, thus, to the stretching of the B1–B1 inter-icosahedral bonds.

Such a division of *o*-B_6_S and *o*-B_6_Se normal modes is consistent with previously reported classification of vibrational modes of α-B_12_ and isostructural boron-rich compounds^22,23,26,27^: the modes involving the whole icosahedron rotations lay in the 100–200 cm^−1^ range, intra-icosahedral modes lay between 550–950 cm^−1^, and inter-icosahedral modes are with wave numbers above 1000 cm^−1^.

The detailed explanation of the bands widths over ~600 cm^−1^ requires additional XRD and Raman studies of *o*-B_6_S and *o*-B_6_Se single crystals (perhaps coupled with low-temperature and high-pressure measurements). Nonetheless, it might be assumed, that some random distortions of B_12_-icosahedral units (not detectable by powder XRD) and, thus, corresponding distortion of the intra- and inter-icosahedral bonds as well as partial occupation of *4 h* sites by S/Se atoms might be the most probable reasons of the observed Raman bands broadening. Earlier, the isotopic ^11^B/^10^B disorder in α-boron was also proposed as a possible reason of the Raman bands broadening^23^.

To conclude, in the present work new boron-rich sulfide *o*-B_6_S and selenide *o*-B_6_Se were synthesized under extreme *p-T* conditions and studied by powder X-ray diffraction and Raman spectroscopy at ambient pressure. With the help of *ab initio* evolutionary crystal structure prediction combined with Rietveld refinement of synchrotron X-ray diffraction data, the crystal structures of the boron-rich chalcogenides were refined. Both phases have orthorhombic symmetry and belong to the same space group *Pmna* (53). The observed Raman bands were assigned to the phonon modes and associated with atomic movements. Elastic properties of new boron-rich chalcogenides were theoretically predicted using various *ab initio* methods.

## Methods

### Experimental

Formation of new boron-rich chalcogenides was first observed in our *in situ* high pressure – high temperature studies of the B–S and B–Se binary systems at BL04B1 beamline, SPring-8 (Japan) and PSICHE beamline, SOLEIL (France). Chemical interaction of elemental boron with sulfur and selenium melts were studied by energy-dispersive X-ray diffraction at pressures up to 11 GPa and temperatures up to 2500 K using SPEED-1500 multianvil press (BL04B1) and Paris-Edinburgh press (PSICHE) using white beam (20–150 keV, bending magnet @ BL04B1; 25–80 keV, wiggler source @ PSICHE).

Based on the information about the most appropriate synthesis conditions and optimal stoichiometries of B:S(Se) reaction mixtures extracted from our synchrotron studies, the new boron-rich chalcogenides have been synthesized at 6.1 GPa and 2700 K in a toroid-type high-pressure apparatus. A design of the high-temperature assembly used in recovery experiments is described elsewhere^32^. The powders of amorphous boron (Grade I ABCR), and sulfur and selenium (both Alfa Aesar, 99.5%) were used as starting materials. Boron nitride capsules (COMPRES) were used to isolate the reaction mixture (B:X molar ratio 5:1) from the graphite heater. The recovered samples were ground in mortar and treated with 3 N nitric acid (ACS, Alfa Aesar) for 20 min at 370 K in order to remove unreacted elements, washed with deionized water and dried at 400 K. The chemical composition of synthesized compounds was studied by energy-dispersive X-ray spectroscopy using scanning electron microscope FEI Quanta 200 F at 10 kV accelerating voltage (see. Fig. S2).

X-ray diffraction study of boron-rich chalcogenides was performed at Swiss-Norwegian Beamline BM01, ESRF^33^. The wavelength of monochromatic beam from a bending magnet was set to 0.6866 Å. X-ray diffraction patterns were collected during 20 s in Debye-Scherrer geometry with rotating quartz-glass capillary using PILATUS 2 M detector. The crystal structure refinement was performed using Maud software^34^; high purity LaB_6_ was used as a standard.

Raman spectra of powder polycrystalline samples were measured in different spatial points at ambient conditions in the 100–2000 cm^−1^ range using Horiba Jobin Yvon HR800 Raman spectrometer; the spectrometer was calibrated using single-crystal cubic Si at room temperature. Unpolarized light from 633-nm line of He-Ne laser (10 µm beam spot) was used for excitation. The measurements were also performed at 473-nm excitation wavelength; no resonant effects and/or significant photoluminescence were observed (Fig. S1).

### Computational details

X-ray diffraction patterns of the newly synthesized phases clearly did not match any previously known phases. Neither their structures, nor the exact chemical compositions were known. Taking into account the starting B:S/Se molar ratios we assumed the probable composition as: B_*x*_S and B_*x*_Se with 5 ≤ *x* ≤ 7. This information was insufficient for the determination of the crystal structures solely from experiment.

We performed variable-composition searches for all stable compounds in the B-S and B-Se systems using the USPEX code^35–37^, which has already demonstrated exceptional predictive power, reliability and efficiency for discovering novel compounds and their crystal structures (e.g.^38–41^.). Searches were performed at the pressure of 20 GPa, the initial population was made of structures containing up to 30 atoms in the primitive unit cell. In each generation there were 60 structures, and calculations were run for 60 generations. All produced structures were carefully relaxed and their enthalpies were computed using the Vienna *ab initio* Simulation Package (VASP)^42^ within the generalized gradient approximation (GGA) of Perdew-Burke-Ernzerhof (PBE)^43^. Total energy was calculated within the framework of projector augmented wave (PAW) method^44^. We used plane wave energy cutoff of 550 eV and Gamma-centered *K*-point mesh with the resolution of 2π·0.06 Å^−1^ for final structural relaxations in USPEX. For mechanical and electronic property calculations, we improved the *K*-point mesh to the resolution of 2π·0.04 Å^−1^. Vickers hardness was estimated using Chen^45^ and Mazhnik-Oganov^46^ models, while the fracture toughness was calculated by Niu-Oganov^47^ and Mazhnik-Oganov^46^ models. The most reliable results should be expected from Mazhnik-Oganov models, and the discrepancy between the used models gives an idea of the results uncertainty.

The Raman spectra of both boron-rich chalcogenides were computed using VASP code with the fully relaxed structure. Firstly, we performed phonon calculation to determine phonon frequencies and normal modes at the Γ-point based on density-functional perturbation theory (DFPT) as implemented in the PHONOPY code. Further DFPT method was used to compute out macroscopic dielectric tensor. And lastly, Raman intensity for each normal mode was obtained by calculating the derivative of the calculated macroscopic dielectric tensor (or polarizability) with respect to the corresponding normal mode coordinate.

At the same time, structural and phonon properties of both boron-rich chalcogenides were also studied using linear combination of atomic orbital (LCAO) calculations based on the hybrid exchange-correlation density functional (DFT)/Hartree-Fock (HF) scheme, which is implemented in CRYSTAL17 code^48^. For boron and sulfur atoms we used all-electron basis sets which were optimized in earlier calculations^49,50^. The core electrons of the selenium atoms were excluded from consideration using the effective core pseudopotential (ECP) with corresponding atomic basis set^50^. The accuracy of the calculation of the bielectronic Coulomb and exchange series is controlled by the set of tolerances, which were taken to be 10^−7^, 10^−7^, 10^−7^, 10^−9^, and 10^−30^, according to the recommendation for hybrid functionals^50^. The Monkhorst-Pack scheme^51^ for an 8 × 8 × 8 k-point mesh in the Brillouin zone was applied. Self-consistent field calculations were performed for hybrid DFT/HF WCGGA-PBE-16% functional^52^. The percentage 16% defines the Hartree-Fock admixture in the exchange part of DFT functional.

The full structure optimization procedure according to the energy minima criterion was performed for both boron-rich chalcogenides. The bulk moduli of both compounds were estimated using routine implemented in CRYSTAL17 code^53^. The unit cell volumes were varied from 95% to 105% of the volume (V_0_) corresponding to the energy minimum (E_0_). The structure optimization was performed at each volume value. The obtained E(V) dependences were fitted to the Birch-Murnaghan equation of state.

The phonon frequencies for both compounds were calculated using the direct (frozen-phonon) method implemented in CRYSTAL17 code^54,55^. Calculation of Raman intensities was performed by using a coupled-perturbed Hartree–Fock/Kohn–Sham approach^55,56^. Raman spectra were constructed by using the transverse optical (TO) modes and by adopting a pseudo-Voigt functional form^54^ with a full width half maximum parameter set to 1. The choice of the broadening was determined according to the criteria to keep maximal possible small intensity bands in theoretical spectrum, which are smeared out while applying higher broadening parameters.

## Supplementary information

Supplementary information.

Supplementary information2.

Supplementary information3.
