# Supplementary material for: Discovery of new boron-rich chalcogenides: orthorhombic B6X (X=S, Se)
Source: Sci Rep. 2020 Jun 9;10:9277. doi: 10.1038/s41598-020-66316-y (PMC7283469; doi:10.1038/s41598-020-66316-y)
Supplement: Supplementary file 3 — Supplementary information3. [file 41598_2020_66316_MOESM3_ESM.doc]

**Discovery of new boron-rich chalcogenides: orthorhombic B_6_X (X = S, Se)**

**Kirill A. Cherednichenko^1^, Vladimir A. Mukhanov^1^, Zhenhai Wang^2,3^, Artem R. Oganov^2,4,5^, Aleksandr Kalinko^6,7^, Iurii Dovgaliuk^8^ and Vladimir L. Solozhenko^1^,***

^1^ LSPM–CNRS, Université Paris Nord, Villetaneuse, 93430, France.

^2^ Skolkovo Institute of Science and Technology, Skolkovo Moscow Region, 143026, Russia.

^3^ Nanjing University of Posts and Telecommunications, Nanjing, Jiangsu 210003, China.

^4^ Moscow Institute of Physics and Technology, Dolgoprudny City, Moscow Region 141700, Russia.

^5^ School of Materials Science, Northwestern Polytechnical University, Xi'an 710072, China.

^6^ Institute of Solid State Physics, University of Latvia, Riga, LV-1063, Latvia.

^7^ Universität Paderborn, Naturwissenschaftliche Fakultät, Paderborn, 33098, Germany.

^8^ European Synchrotron Radiation Facility, Grenoble, 38043, France.

* vladimir.solozhenko@univ-paris13.fr

**Table S1** The atomic structure (first predicted by USPEX) of the orthorhombic boron-rich sulfide and selenide retrieved from Rietveld refinement and *ab initio* geometry optimizations (VASP and CRYSTAL17).

|  | Phase | Atom label (Wyck.) | *x* | *y* | *z* | *B_iso_*, Å^2^ | Site occupancy |
| --- | --- | --- | --- | --- | --- | --- | --- |
| *Experimental* | *o*-B_6_S | S1 (*4h*) | 0.0000 | 0.1863(1) | 0.8681(1) | 1.401(3) | 0.925(3) |
|  |  | B1 (*4h*) | 0.0000 | 0.1578(6) | 0.4835(4) | 2.05(8) | 1.0^f^ |
|  |  | B2 (*8i*) | 0.8385(4) | 0.3565(4) | 0.3358(3) | 2.23(6) | 1.0^f^ |
|  |  | B3 (*4h*) | 0.0000 | 0.3454(6) | 0.6665(5) | 2.39(8) | 1.0^f^ |
|  |  | B4 (*8i*) | 0.2591(5) | 0.3511(5) | -0.0432(3) | 2.34(8) | 1.0^f^ |
|  | *o*-B_6_Se | Se1 (*4h*) | 0.0000 | 0.1695(1) | 0.8663(9) | 0.31(2) | 0.952(5) |
|  |  | B1 (*4h*) | 0.0000 | 0.1605(12) | 0.4803(7) | 1.45(18) | 1.0^f^ |
|  |  | B2 (*8i*) | 0.8402(5) | 0.3638(8) | 0.3363(6) | 0.83(14) | 1.0^f^ |
|  |  | B3 (*4h*) | 0.0000 | 0.3493(12) | 0.6623(8) | 0.39(14) | 1.0^f^ |
|  |  | B4 (*8i*) | 0.2638(7) | 0.3531(11) | -0.0396(5) | 0.58(13) | 1.0^f^ |
| VASP | *o*-B_6_S | S1 (*4h*) | 0.0000 | 0.1879 | 0.8698 | — | 1.0 |
|  |  | B1 (*4h*) | 0.0000 | 0.1631 | 0.4829 | — | 1.0 |
|  |  | B2 (*8i*) | 0.8417 | 0.3628 | 0.3361 | — | 1.0 |
|  |  | B3 (*4h*) | 0.0000 | 0.3491 | 0.6668 | — | 1.0 |
|  |  | B4 (*8i*) | 0.2598 | 0.3521 | -0.0444 | — | 1.0 |
|  | *o*-B_6_Se | Se1 (*4h*) | 0.0000 | 0.1701 | 0.8677 | — | 1.0 |
|  |  | B1 (*4h*) | 0.0000 | 0.1644 | 0.4812 | — | 1.0 |
|  |  | B2 (*8i*) | 0.8424 | 0.3672 | 0.3358 | — | 1.0 |
|  |  | B3 (*4h*) | 0.0000 | 0.3511 | 0.6608 | — | 1.0 |
|  |  | B4 (*8i*) | 0.2659 | 0.3535 | -0.0418 | — | 1.0 |
| CRYSTAL17 | *o*-B_6_S | S1 (*4h*) | 0.0000 | 0.1851 | 0.8691 | — | 1.0 |
|  |  | B1 (*4h*) | 0.0000 | 0.1627 | 0.4827 | — | 1.0 |
|  |  | B2 (*8i*) | 0.8413 | 0.3618 | 0.3361 | — | 1.0 |
|  |  | B3 (*4h*) | 0.0000 | 0.3487 | 0.6666 | — | 1.0 |
|  |  | B4 (*8i*) | 0.2589 | 0.3515 | -0.0445 | — | 1.0 |
|  | *o*-B_6_Se | Se1 (*4h*) | 0.0000 | 0.1669 | 0.8667 | — | 1.0 |
|  |  | B1 (*4h*) | 0.0000 | 0.1637 | 0.4811 | — | 1.0 |
|  |  | B2 (*8i*) | 0.8418 | 0.3655 | 0.3358 | — | 1.0 |
|  |  | B3 (*4h*) | 0.0000 | 0.3505 | 0.6609 | — | 1.0 |
|  |  | B4 (*8i*) | 0.2641 | 0.3526 | -0.0421 | — | 1.0 |

^f^ the atom site occupancies were fixed to 1.0;

**Table S2.** The frequencies of the experimentally observed Raman bands at 633 nm excitation wavelength (ω_0_) and Raman active modes predicted by VASP and CRYSTAL17 (ω*_t_*^V^ and ω*_t_*^C^), respectively. The overlapped bands groups observed in experimental Raman spectra are presented by the corresponding frequencies regions; "—" signs indicate the absence of the bands/bands groups in the experimental Raman spectra.

| ***o*-B_6_S** | | | | | | | | | | | | | |
| --- | --- | --- | --- | --- | --- | --- | --- | --- | --- | --- | --- | --- | --- |
| ω_0_, cm^-1^ | ω*_t_*^V^, cm^-1^ | ω*_t_*^C^, cm^-1^ | Modes | | ω_0_, cm^-1^ | ω*_t_*^V^, cm^-1^ | ω*_t_*^C^, cm^-1^ | Modes | | ω_0_, cm^-1^ | ω*_t_*^V^, cm^-1^ | ω*_t_*^C^, cm^-1^ | Modes |
| 305.47 | 303.55 | 309.33 | B_1g_ | | 618.86 | 616.91 | 634.64 | A_g_ | | 780.02 | 781.84 | 802.09 | A_g_ |
| 307-319 | 307.25 | 317.66 | A_g_ | | — | 640.41 | 660.56 | B_3g_ | | 809.17 | 802.65 | 824.64 | B_3g_ |
|  | 308.65 | 318.39 | B_2g_ | |  | 655.42 | 673.72 | B_2g_ | | — | 835.46 | 852.28 | B_2g_ |
| 334.46 | 331.40 | 340.95 | B_3g_ | | 673-706 | 682.21 | 698.92 | B_3g_ | |  | 878.89 | 896.82 | B_1g_ |
| 347.05 | 338.70 | 350.08 | A_g_ | |  | 689.44 | 707.51 | A_g_ | | 939-1079 | 952.53 | 977.18 | B_1g_ |
| 373.25 | 363.94 | 378.07 | B_2g_ | |  | 694.10 | 712.08 | B_1g_ | |  | 950.51 | 978.60 | A_g_ |
| 379.25 | 376.60 | 382.20 | B_1g_ | |  | 694.85 | 712.79 | B_2g_ | |  | 964.46 | 988.51 | B_3g_ |
| 435.25 | 429.62 | 437.67 | B_3g_ | | 708-740 | 712.47 | 734.36 | A_g_ | |  | 962.88 | 989.83 | B_2g_ |
| 466.54 | 460.38 | 470.99 | A_g_ | |  | 723.37 | 742.55 | B_1g_ | |  | 992.15 | 1022.98 | B_2g_ |
| 470-490 | 464.96 | 475.70 | B_1g_ | |  | 730.64 | 751.44 | B_2g_ | |  | 992.92 | 1023.62 | A_g_ |
|  | 483.37 | 488.10 | B_3g_ | | 742-772 | 745.54 | 766.73 | A_g_ | |  | 994.05 | 1025.61 | B_3g_ |
| 496.15 | 488.92 | 498.44 | B_2g_ | |  | 751.81 | 768.42 | B_3g_ | |  | 1000.98 | 1030.45 | A_g_ |
| 566.72 | 564.98 | 577.31 | B_1g_ | |  | 755.86 | 772.11 | B_1g_ | |  | 1015.79 | 1052.08 | B_2g_ |
| 581.77 | 583.30 | 593.99 | B_2g_ | |  | 763.57 | 784.87 | B_2g_ | |  | 1016.45 | 1053.83 | A_g_ |
| ***o*-B_6_Se** | | | | | | | | | | | | | |
| ω_0_, cm^-1^ | ω*_t_*^V^, cm^-1^ | ω*_t_*^C^, cm^-1^ | Modes | ω_0_, cm^-1^ | | ω*_t_*^V^, cm^-1^ | ω*_t_*^C^, cm^-1^ | Modes | ω_0_, cm^-1^ | | ω*_t_*^V^, cm^-1^ | ω*_t_*^C^, cm^-1^ | Modes |
| 192-201 | 191.69 | 197.87 | B_2g_ | — | | 612.38 | 632.73 | B_2g_ | 824.84 | | 824.75 | 843.56 | B_2g_ |
|  | 192.52 | 198.57 | A_g_ | — | | 618.65 | 640.87 | B_3g_ | — | | 857.69 | 876.31 | B_1g_ |
| 205.18 | 202.36 | 208.23 | B_1g_ | 641-679 | | 647.48 | 665.89 | A_g_ | 921.87 | | 907.53 | 940.63 | A_g_ |
| 210.06 | 206.61 | 213.17 | B_3g_ |  |  | 652.7696 | 669.76 | B_3g_ | 933-1028 | | 930.32 | 960.39 | B_3g_ |
| 248.82 | 243.52 | 251.13 | A_g_ |  |  | 661.47 | 680.76 | B_1g_ |  |  | 946.73 | 965.96 | B_1g_ |
| 260.55 | 255.44 | 263.67 | B_2g_ |  |  | 668.24 | 686.26 | B_2g_ |  |  | 939.27 | 968.32 | B_2g_ |
| 347.69 | 344.68 | 349.24 | B_1g_ | 682-784 | | 686.33 | 704.41 | B_1g_ |  |  | 931.71 | 973.02 | A_g_ |
| 417.56 | 410.26 | 418.81 | B_3g_ |  |  | 692.65 | 716.87 | A_g_ |  |  | 961.14 | 984.44 | B_2g_ |
| 429.24 | 422.95 | 433.59 | A_g_ |  |  | 702.14 | 726.48 | B_2g_ |  |  | 949.41 | 985.52 | B_3g_ |
| 449.07 | 441.87 | 450.73 | B_1g_ |  |  | 728.38 | 749.73 | B_3g_ |  |  | 975.69 | 1001.11 | A_g_ |
| 468-476 | 465.31 | 475.43 | B_3g_ |  |  | 731.63 | 751.82 | A_g_ |  |  | 984.30 | 1013.15 | A_g_ |
|  | 468.69 | 475.86 | B_2g_ |  |  | 742.69 | 762.78 | B_1g_ |  |  | 990.05 | 1017.43 | B_2g_ |
| 565.42 | 551.84 | 568.31 | B_1g_ |  |  | 747.03 | 763.54 | B_2g_ |  | |  |  |  |
| 572.88 | 567.14 | 578.41 | B_2g_ |  |  | 770.66 | 791.88 | A_g_ |  | |  |  |  |
| 594.62 | 591.37 | 610.71 | A_g_ |  |  | 773.65 | 796.31 | B_3g_ |  | |  |  |  |


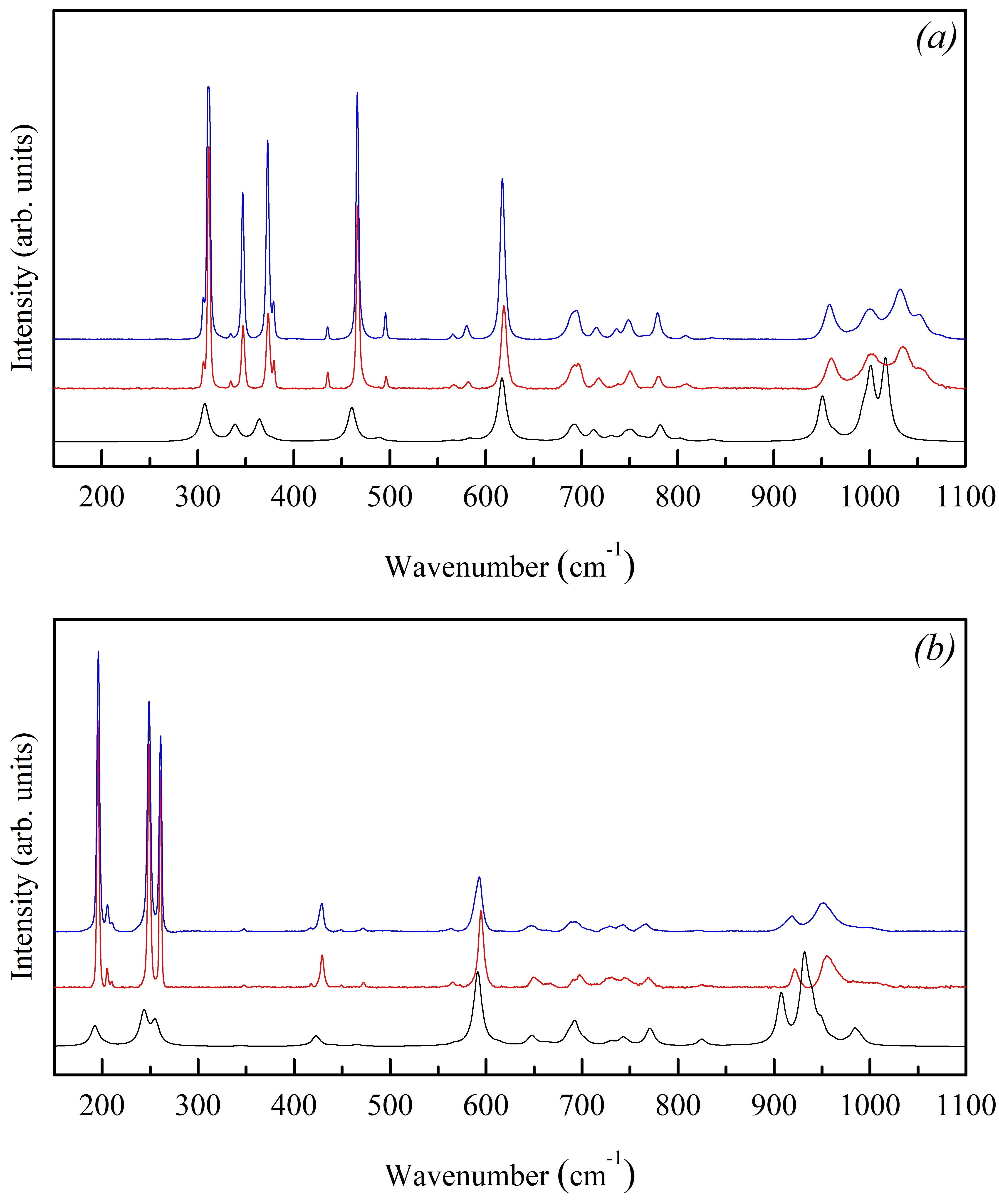


**Fig. S1**. Experimental (red and blue) and VASP-calculated (black) Raman spectra of *o*-B_6_S (*a*) and *o*-B_6_Se (*b*) at ambient conditions. The experimental Raman spectra were measured at two different excitation wavelengths: 473 nm (blue) and 633 nm (red).


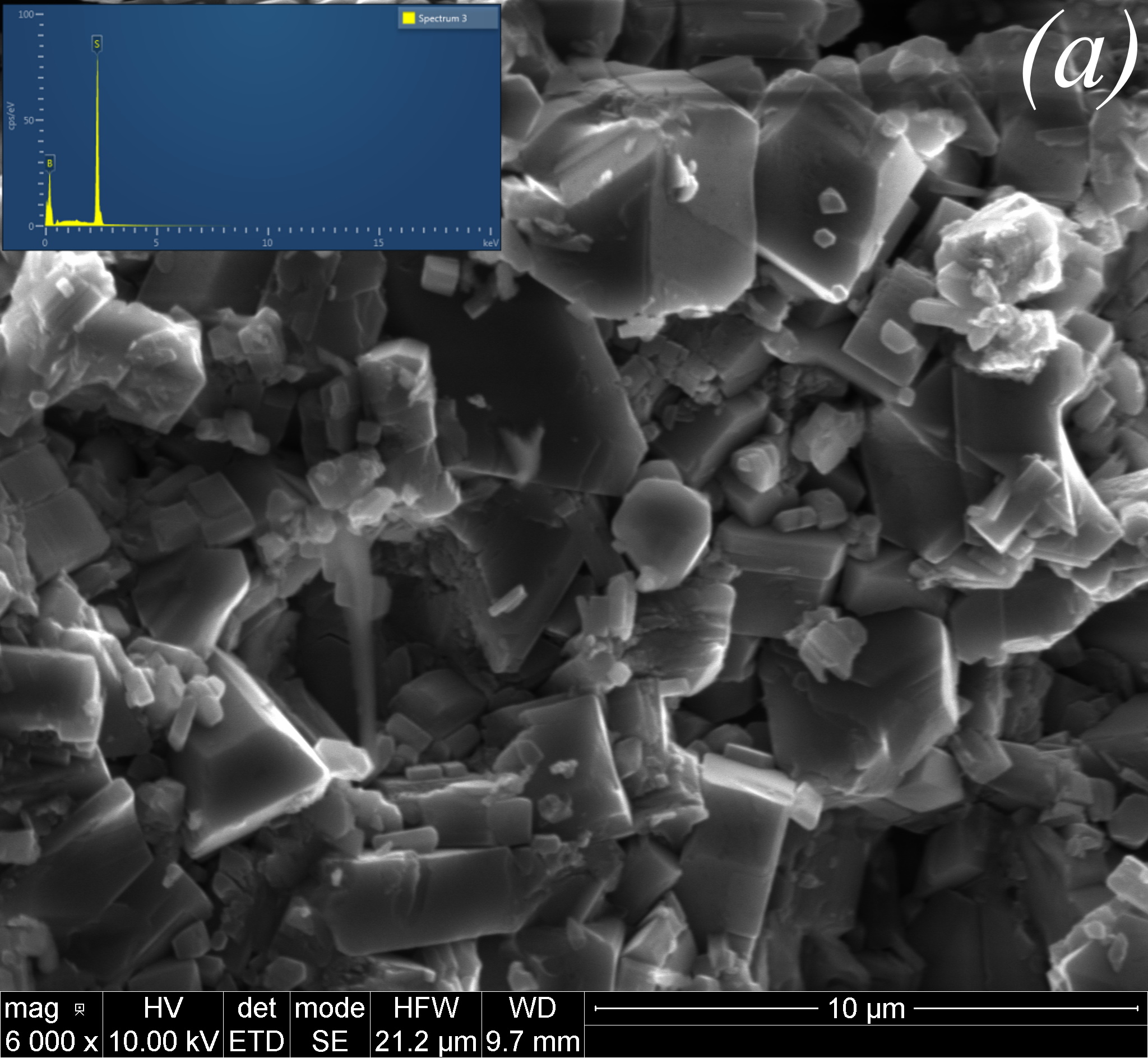


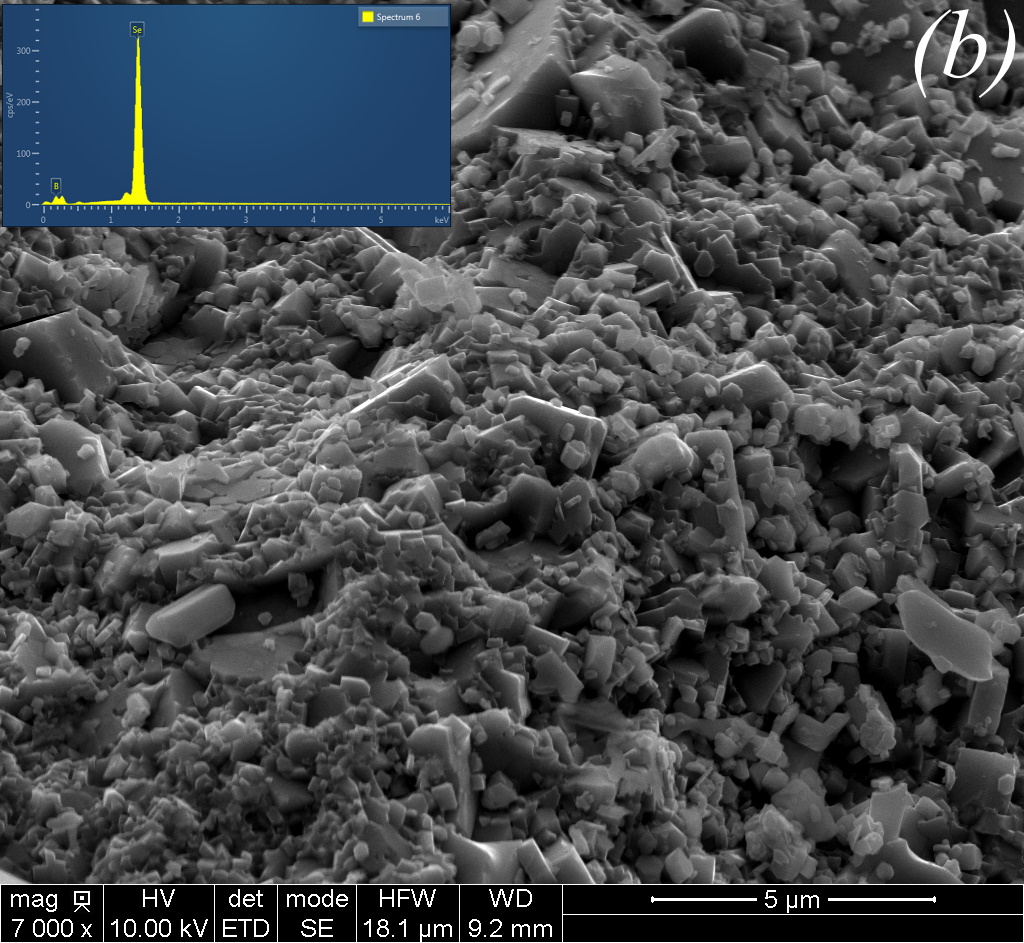


**Fig. S2**. SEM images of *o*-B_6_S (*a*) and *o*-B_6_Se (*b*); the insets present the corresponding EDX spectra.
